# Design, Fabrication, Characterization, and Simulation of AlN-Based Piezoelectric Micromachined Ultrasonic Transducer for Sonar Imaging Applications

**DOI:** 10.3390/mi15060781

**Published:** 2024-06-13

**Authors:** Wenxing Chen, Shenglin Ma, Xiaoyi Lai, Zhizhen Wang, Hui Zhao, Qiang Zha, Yihsiang Chiu, Yufeng Jin

**Affiliations:** 1 Department of Mechanical & Electrical Engineering, Xiamen University, Xiamen 361005, China; 2 The Fifth Research Laboratory, Shanghai Marine Electronic Equipment Research Institute, Shanghai 201108, China; 3 Nanofabrication Facility, Suzhou Institute of Nano-Tech and Nano-Bionics, Chinese Academy of Sciences, Suzhou 215123, China; 4 School of Electronic and Computer Engineering, Peking University Shenzhen Graduate School, Shenzhen 518055, China

**Keywords:** AlN PMUT, receiving sensitivity, acoustic characteristics simulation model

## Abstract

To address the requirements of sonar imaging, such as high receiving sensitivity, a wide bandwidth, and a wide receiving angle, an AlN PMUT with an optimized ratio of 0.6 for the piezoelectric layer diameter to backside cavity diameter is proposed in this paper. A sample AlN PMUT is designed and fabricated with the SOI substrate-based bulk MEMS process. The characterization test result of the sample demonstrates a −6 dB bandwidth of approximately 500 kHz and a measured receiving sensitivity per unit area of 1.37 V/μPa/mm^2^, which significantly surpasses the performance of previously reported PMUTs. The −6 dB horizontal angles of the AlN PMUT at 300 kHz and 500 kHz are measured as 68.30° and 54.24°, respectively. To achieve an accurate prediction of its characteristics when being packaged and assembled in a receive array, numerical simulations with the consideration of film stress are conducted. The numerical result shows a maximum deviation of ±7% in the underwater receiving sensitivity across the frequency range of 200 kHz to 1000 kHz and a deviation of about 0.33% in the peak of underwater receiving sensitivity compared to the experimental data. By such good agreement, the simulation method reveals its capability of providing theoretical foundation for enhancing the uniformity of AlN PMUTs in future studies.

## 1. Introduction

The ultrasonic transducer plays an important role in a sonar imaging system as it helps to realize the mutual conversion of electrical and acoustic signals. For sonar imaging equipment, ultrasonic transducers are usually fabricated with bulk Piezoceramics and work in thickness vibration mode [1]. Although this kind of ultrasonic transducers have a long history and are developed as an industry with high technological maturity, there are some limitations. For example, it features a large volume size, high power consumption, requirements for precision machining and assembly, and a high acoustic impedance with requirements for an additional acoustic impedance matching layer [2]. In addition, the resonant frequency of the ultrasonic transducer in the thickness mode increases as the thickness of the piezoelectric material decreases, which means there is an increase in difficulty regarding machining and assembly to achieve a high resonant frequency. For example, for a resonant frequency of 500 kHz, the thickness of the piezoelectric material should be reduced to less than 3 mm. With the rapid development of micro-electro-mechanical systems (MEMSs) technology, micro-ultrasonic transducers (MUTs) have emerged. As MUTs have a small size, batch mode production, and CMOS process compatibility and can easily be arrayed with advanced package technology, many researchers have explored its applications to replace traditional ultrasonic transducers. According to the principle of operation, micro-ultrasonic transducers can be classified into two types: capacitive micro-ultrasonic transducers (CMUTs) and piezoelectric micro-ultrasonic transducers (PMUTs) [3,4,5]. Compared to CMUTs, PMUTs have the advantages of a wide resonant frequency range coverage and independence on DC bias voltage [6,7]. Yang D et al. [8] proposed a piezoelectric AlN PMUT with a resonance frequency of 986 kHz, achieving an underwater receiving sensitivity of −178 dB (re: 1 V/μPa). To explore the application of underwater imaging, Yao, Y et al. [1] proposed a PMUT which exhibits a transmit sensitivity of 146.59 dB (re: 1 μPa/V) and a receive sensitivity of −173.70 dB. To explore the application of underwater acoustic communication, Zhao, J et al. [9] proposed a transducer fabricated from piezoelectric ceramic with a maximum transmit voltage response of 152.1 dB (re: 1 μPa·m/V) and a receiving sensitivity of 182.5 dB. When reviewing published studies, it can be concluded that although the transmit sensitivity of an AlN PMUT is much lower than that of traditional bulk transducers, their underwater receiving sensitivities are comparable. In addition, compared with PZT, AlN is free of toxic lead and is compatible with CMOS technology. Although its piezoelectric coefficient is one order of magnitude smaller than that of PZT, its dielectric constant is two orders of magnitude smaller, and theoretically, the receiving sensitivity of an AlN PMUT is one order of magnitude higher than that of a PZT PMUT [6]. Therefore, AlN PMUTs have good potential to replace bulk transducers in sonar imaging receiver arrays.

For sonar imaging equipment, tens of thousands of channels of transducers are desired in a receiver array. This not only imposes a high demand on the receiving sensitivity, bandwidth, and receiving angle, but also on the uniformity across all transducers to ensure a good overall performance. However, AlN PMUTs based on the MEMS process are sensitive to stress due to their inherent characteristics resulting from the suspended structure made of multi-layered thin films. Residual stress in an AlN film still poses a significant challenge [10]. Additionally, considering subsequent steps during its packaging and assembly, when different functional materials such as acoustic matching material and water sealing are used, the difficulty of controlling stress and designing a PMUT further increases. In order to mitigate the influence of residual stress, novel structures have been proposed, such as the PMUTs with V-shaped springs proposed by Chen, X et al. [11] and the tapered cantilever cluster PMUT proposed by Gong, Y et al. [12], although both structures are more suitable for applications in air. M Olfatnia et al. [13] and Firas Sammoura et al. [14] have investigated the influence of residual stress on the vibration behavior characteristics and displacement sensitivity of PZT piezoelectric thin films. In 2020, Glenn Ross et al. [15] used X-ray diffraction (XRD) technology to measure the residual stress in PMUTs at different positions on wafers. The results show compressive stress ranges from −357 MPa to −56 MPa radially on the wafer and a shift in the first-order resonance frequency from 400 kHz to 600 kHz. The influence brought by residual stress is too significant to be neglected; therefore, a simulation method factoring in the effects of stress on the characteristics of PMUTs should be developed to make a high-precision prediction of the performance of PMUTs in sonar receiver arrays.

This paper, which aims to develop a PMUT-based receiving array for 3D sonar imaging operating in the range from 200 to 700 kHz, an AlN PMUT is designed, fabricated, and tested, and a simulation method is established to achieve the high-precision prediction of performance parameters such as the operating frequency, receiving sensitivity, and bandwidth underwater.

## 2. Design of AlN PMUT

The resonant frequency of the PMUT is largely dependent on the structure designs and the residual stresses within the films. The resonant frequency of the PMUT operating in air can be expressed as follows [16]:

(1) $$f=\frac{1}{2\pi }\sqrt{\frac{\alpha^{2}D}{\mu d^{4}}\left(\alpha^{2}+\kappa^{2}\right)}$$

(2) $$\kappa^{2}=\frac{Td^{2}}{D}$$

(3) $$T=\sum_{n=1}^{N}\sigma_{i}t_{i}$$

where $\alpha^{2}$ represents the non-dimensional frequency parameters for the first resonant frequency, *D* is the flexural rigidity, d is the diameter of cavity, μ is the mass per unit area, *T* is the residual tension, $\sigma_{i}$ is the tensile residual stress of the *i*-th layer, and $t_{i}$ is the thickness of the *i*-th layer.

When the PMUT is working in water, the resonant frequency in water can be expressed as follows [1]:

(4) $$f_{water}=\frac{f_{air}}{\sqrt{1+\frac{0.335d\rho_{water}}{\mu }}}$$

where $\rho_{water}$ is the density of water.

According to Equations (1)–(4), the cavity diameter is determined to be 350 μm, which has a theoretical first-order resonant frequency of 310.5 kHz in water, factoring in the residual stress of the AlN layer of 500 MPa, which is the worst case based on the existing literature [17].

**Figure 1:**
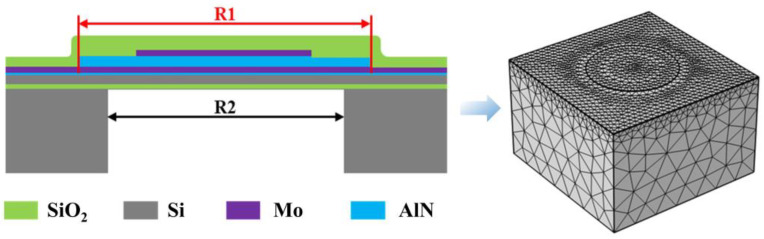
Schematic of PMUT cross section and simulation model.

**Figure 2:**
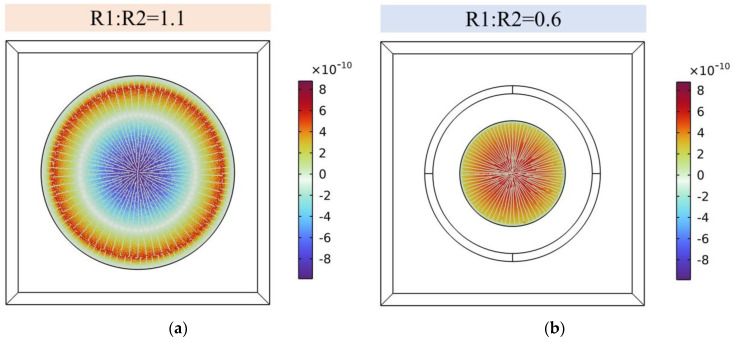
Potential simulation results of PMUT with different piezoelectric dimensions, listed as follows: (**a**) R1:R2 = 1.1; (**b**) R1:R2 = 0.6.

To further analyze the properties of the designed PMUTs, a 3D model of a PMUT with a cavity diameter of 350 µm is established, as shown in Figure 1. The material parameters and dimensions of each layer are shown in Table 1. In Figure 2a, it can be seen that the circular piezoelectric film shows a very obvious zero-potential ring (white) when the ratio of the piezoelectric layer diameter (R1) to the cavity diameter (R2) is 1.1. The zero-potential ring is about 60% of the diameter of the film, while opposite potentials are inside and outside of the circle, respectively. Based on the distribution of the potential on the PMUT, in order to achieve better device performance, many scholars [14,18,19] have proposed optimized sizes of the upper electrode, which was usually designed to be 0.6–0.7 of the cavity’s diameter. However, due to the fact that the size of the piezoelectric layer is larger than the radius of the cavity, the constraints of the piezoelectric layer are solidly supported constraints all around, which leads to a large anchor loss in the piezoelectric film. In order to reduce anchor loss, the size of the piezoelectric layer is optimally designed. When the ratio of the piezoelectric layer diameter (R1) to the cavity diameter (R2) is 0.6, the potential distributions of its piezoelectric film are all positive, as shown in Figure 2b, which indicates that minimized anchor loss is achieved without decreasing the PMUT’s potential.

**Figure 3:**
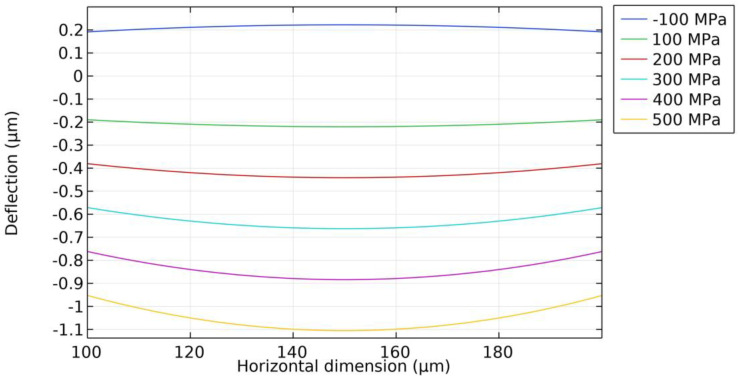
Simulation results of deflection of suspended stacked film at center with AlN piezoelectric layer under different residual stress conditions.

Residual stress is present in thin films such as Mo electrodes and AlN piezoelectric layers. The thickness of the top and bottom Mo electrodes is only 0.2 µm, which is approximately 10% of the piezoelectric layer’s thickness. According to [20], the residual stress in Mo electrodes is about 400 MPa. When the residual stress of the AlN piezoelectric layer is 500 MPa, according to Equation (3), the residual stress contribution from the Mo electrodes to the overall residual stress is roughly 8% of that from the AlN piezoelectric layer. Consequently, to simplify the analysis, focus is placed on investigating the effect of residual stress in the piezoelectric layer on the PMUT. In order to evaluate the effect of different residual stresses, the initial deflection of the PMUT is simulated at first. The ratio of the diameter of the piezoelectric layer to the cavity’s diameter is 0.6, a fixed constraint is set on the bottom silicon substrate, the residual stress of the AlN piezoelectric layer is set from −100 MPa to 500 MPa with a step size of 100 MPa, and the simulation results of the deflection in the direction of the *Z*-axis at the center of the PMUT film are shown in Figure 3. Under the condition that the residual stress of the AlN piezoelectric layer is tensile stress (>0 MPa), the film is deformed in a downward depression, and the deflection of the film increases as the residual tensile stress increases. While under the condition that the residual stress of the AlN piezoelectric layer is compressive stress (<0 MPa), the film is bent in an upward convex direction, and compressive stress causes the film to be in the yielding state, which is prone to lead to the film breaking and other situations.

**Figure 4:**
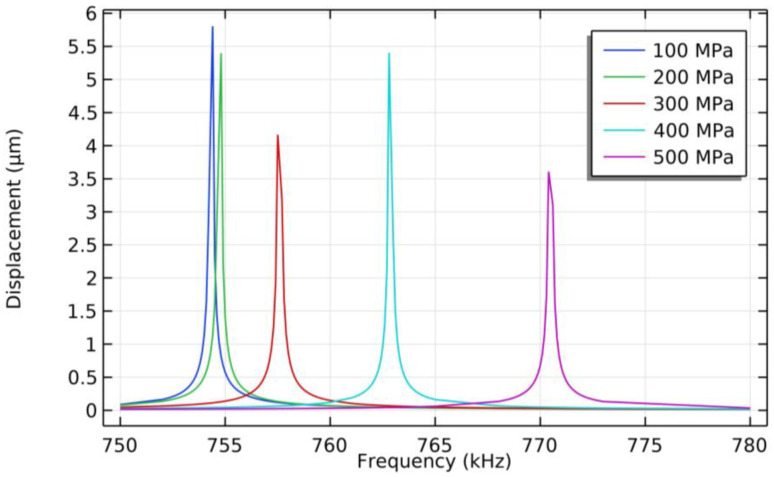
Resonance frequency simulation results of PMUT with AlN piezoelectric layer under different residual stress conditions.

Then, the effect of residual stresses on the PMUT’s resonance frequency is evaluated. The residual stresses of the AlN piezoelectric layer are set to range from 100 MPa to 500 MPa (with step size of 100 MPa), the frequency sweep ranges from 750 kHz to 780 kHz (with step size of 0.1 kHz), and the displacement of the center of the PMUT film in the Z-direction is shown in Figure 4. As the residual tensile stress of the AlN piezoelectric layer increases, the resonance frequency of the PMUT increases. The resonance frequency of the PMUT is 770.4 kHz when the residual tensile stress in the AlN piezoelectric layer is 500 MPa. With Equation (1), the first-order resonant frequencies of a PMUT can be obtained as 771.39 kHz when the residual stress of the AlN layer is 500 MPa, which is consistent with the simulated value.

## 3. Fabrication of AlN PMUT

**Figure 5:**
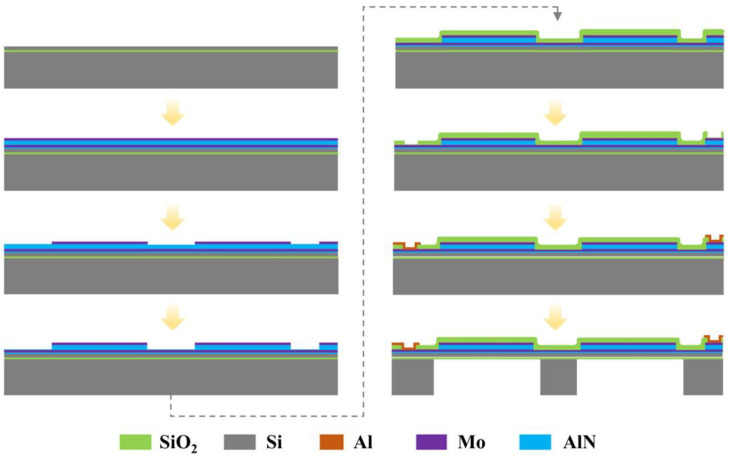
The process flow of the PMUTs.

The fabrication processes are schematically shown in Figure 5. An 8-inch-high resistivity SOI wafer was chosen as the substrate to fabricate the AlN PMUTs, consisting of a Si device layer of 3 μm, a buried oxide layer of 1 μm, and a bulk silicon layer of 650 μm from top to bottom. Firstly, a sandwich structure of Mo/AlN/Mo is deposited on the SOI wafer surface. Then, the top layer of Mo as the electrode is patterned by ion beam etching (IBE). The AlN layer is patterned by inductively coupled plasma (ICP) etching. Next, a SiO_2_ layer of 1 μm in thickness is deposited and patterned with RIE to expose both layers of Mo. After that, an Al layer of 1 μm in thickness is deposited to contact Mo by sputtering and is patterned to form Pads through the lift-off process. Finally, the backside cavity beneath the sandwich structure of Mo/AlN/Mo is formed with the Bosch process.

**Figure 6:**
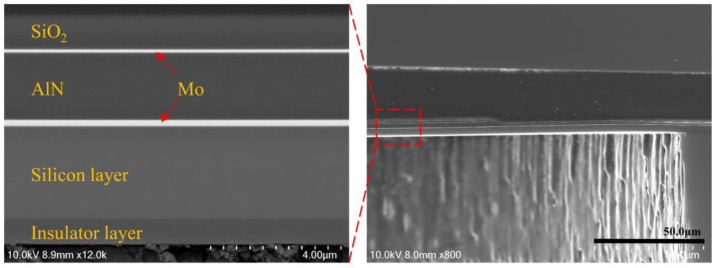
SEM image of PMUT cross section.

**Figure 7:**
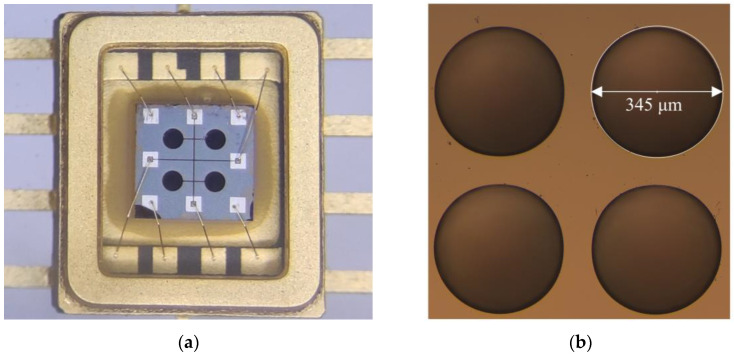
(**a**) Optical microscope image of PMUTs. (**b**) Optical microscope image of cavity.

Figure 6 shows the cross section of AlN PMUT. The measured thickness dimensions are listed as follows: 1 μm for the silica insulating layer, 2 μm for the AlN, 1 μm for the buried oxygen layer, 3 μm for the device layer, 0.2 μm for the lower electrode, and 0.14 μm for the upper electrode. Figure 7a shows the picture of the circular array of 2 × 2 PMUTs, in which a total of four PMUTs are connected in parallel while each PMUT has the same structural dimensions. Figure 7b shows that the diameter of the PMUT back cavity is measured to be 345 μm.

## 4. The Characteristic Measurement of the AlN PMUTs

### 4.1. Resonant Frequency Test

**Figure 8:**
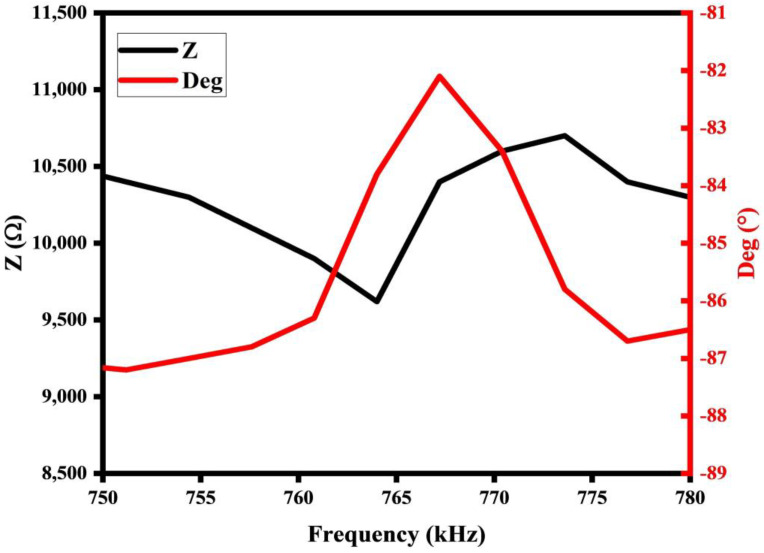
Measurements of resonant frequency of PMUTs in air.

The resonant frequency of the PMUT in air was tested using an impedance analyzer (LCR Meter 6630, MICROTEST Inc., New Taipei City, China), and the results are shown in Figure 8. The frequency setting was scanned from 600 kHz to 1 MHz, and the phase curve (red) peaked at 767.2 kHz, indicating that the first-order resonance frequency of the PMUT in air was 767.2 kHz. The impedance curve has a trough at 764 kHz with a resistance value of 9620 Ω and a peak at 773.6 kHz with a resistance value of 10,700 Ω.

**Figure 9:**
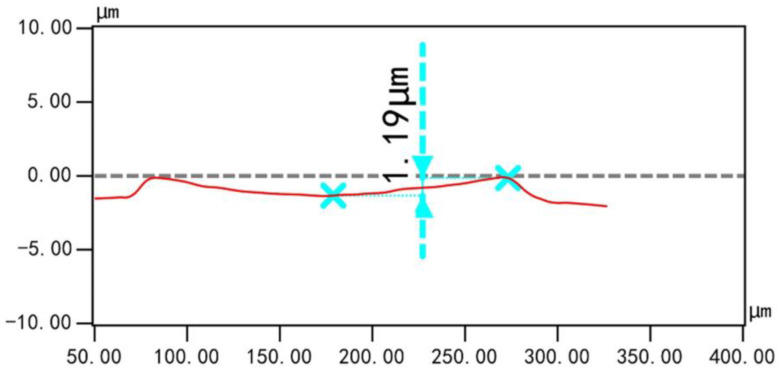
The measurement results of deflection at the center of the film.

The deflection of the PMUT was measured using a 3D laser scanning confocal microscope (VK-X1000, KEYENCE CORPORATION, Taipei City, China), and the test result is shown Figure 9. The deflection at the center of the PMUT film was 1.19 µm, and the film was deformed in a downward concave direction. When combining Figure 3 and Figure 4, the deflection measurements match the deflection prediction for the 500 MPa stress case (1.1 µm) with an error of 0.09 µm, and the PMUT resonance frequency measurements also match the resonance frequency prediction for the 500 MPa stress case (770.4 kHz) with an error of 3.2 kHz, and it can be derived that the PMUT film’s residual stress is 500 MPa.

To evaluate the quality of ultrasonic transducers, electromechanical coupling coefficients are used to quantify such indicators, defined as follows:

(5) $${k_{eff}}^{2}=\frac{{f_{p}}^{2}-{f_{s}}^{2}}{{f_{p}}^{2}}$$

where *f_s_* and *f_p_* are the frequencies at the minimum and the maximum impedances, respectively. Therefore, the electrical coupling factor is calculated to be 2.47%.

### 4.2. Measurement of PMUT’s Underwater Acoustic Properties

**Figure 10:**
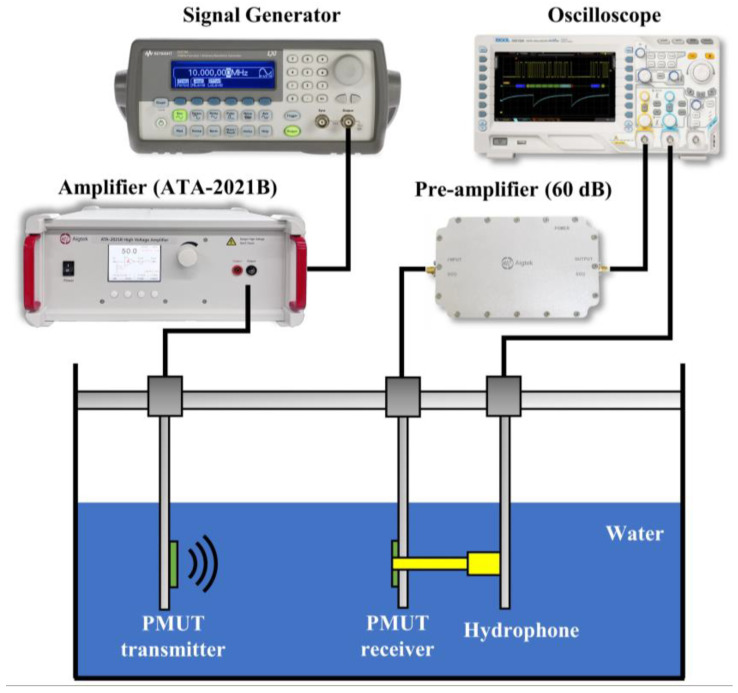
A schematic diagram of the test system for PMUT underwater receiving sensitivity.

**Figure 11:**
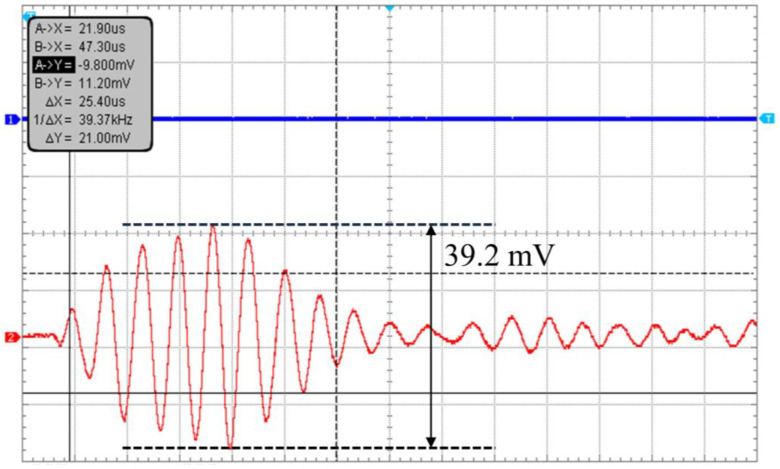
Measurement results of PMUT receiving signals at 300 kHz.

In order to measure the underwater receiving sensitivity of the PMUTs, a commercial standard hydrophone from a research institute was used. At the same time, the PMUT previously developed by our team was used as a transmitter, and the standard hydrophone transducer (RHS-2#, Shanghai Marine Electronic Equipment Research Institute) and the proposed PMUTs as receivers were placed approximately 3.6 cm away from the transmitter to receive ultrasound sequentially, as shown in Figure 10. The signal generator output a 1 Vpp, 5-pulse sinusoidal signal, which was amplified 60 times by a power amplifier (ATA-2021B, Aigtek Ltd., Xi’an, China) to excite the PMUT transmitter to emit acoustic waves. The wave propagated through the water medium and was caught by the PMUT receiver as well as the surface of the hydrophone. The peak-to-peak values of the hydrophone and PMUT receiver voltages were read and analyzed on an oscilloscope. Figure 11 shows the red receiving signal from the PMUT receiver with a peak voltage of 39.2 mV at 300 kHz. The receiving sensitivity of the PMUT is calculated as follows:

(6) $$S_{R}=S_{Hpp}+20lg\frac{V_{R}}{V_{Hpp}}$$

where *S_Hpp_* is the sensitivity of the hydrophone, *V_R_* is the voltage peak-to-peak value of the received signal of the PMUT, and *V_Hpp_* is the voltage peak-to-peak value of the received signal of the hydrophone.

### 4.3. Receiving Angle of PMUT

**Figure 12:**
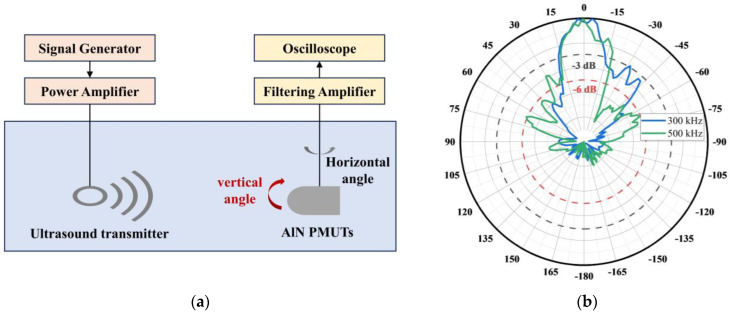
(**a**) A schematic diagram of the measurement system for the receiving angle. (**b**) Measurements of the horizontal angle of the PMUTs.

The receiving angle of the PMUT can be generally divided into horizontal and vertical angles. The measurement principle of the PMUT’s receiving angle is shown in Figure 12a. Output pulse signals are generated by the signal generator, and they are amplified by the power amplifier, which stimulates the sound source to emit sound waves. Keeping the position and direction of the sound source transducer fixed, the transducer is rotated in a certain step in a horizontal or vertical direction, the peak-to-peak value of the output voltage of the transducer is recorded in different directions, and then the receiving sensitivities of the transducer can be calculated according to the calibration data of the sound source. Based on the calculated sensitivities, the received directivity diagrams in horizontal and vertical directions can be plotted. The PMUT in this paper is center-symmetric, which means the horizontal and vertical angles can be considered to be basically equal.

For potential target application to 3D sonar imaging, the transducer transmitter array is designed to operate at frequencies of 300 kHz and 500 kHz, so the PMUT chip was tested for receiving angles at these frequencies. The PMUT was fixed on a rotatable fixture and submerged in water after packaging. The receiving angles of the PMUT at 300 kHz and 500 kHz were tested, respectively, and the test results are shown in Figure 12b. According to the test results, it is observed that the PMUT has a −3 dB horizontal angle of 31.45°, a −6 dB horizontal angle of 68.30° at 300 kHz, a −3 dB horizontal angle of 34.11°, and a −6 dB horizontal angle of 54.24° at 500 kHz. The horizontal angle is improved by two orders of magnitude compared to conventional ultrasonic transducers applied in 3D mapping [21].

## 5. Simulation of PMUTs Underwater

The receiving sensitivity *S_rx_* of a single PMUT is defined as follows [22]:

(7) $$S_{rx}=\frac{{GV}_{rx}}{P_{in}}=\frac{GA_{eff}\;\;}{\;\;\eta \;}\frac{\;\;\eta^{2}Z_{ele}}{\;\;\;{\;\eta }^{2}Z_{ele}+Z_{tot}}$$

where *Z_ele_* is the electrical impedance of a single PMUT, G is the gain of the pre-amplifier, and *Z_tot_* is the sum of the mechanical and acoustic impedance of a single PMUT. When there are *N* PMUTs connected in parallel to form PMUTs, the receiving sensitivity *S_RX_* of the PMUTs is:

(8) $$S_{RX}=\frac{{GV}_{RX}}{P_{in}}=\frac{G{NA}_{eff}\;\;}{\;\;\eta \;}\frac{\;\;\eta^{2}Z_{ele}/N}{\;\;\;{\;\eta }^{2}Z_{ele}/N+Z_{tot}/N}$$

The receiving sensitivity of the PMUTs is proportional to the number of parallel connections, which means that the receiving sensitivity can be improved by increasing the number of parallel connections of PMUTs in a certain area, thus increasing the filling factor. During the subsequent acoustic characteristic simulations, once a single PMUT is simulated, the acoustic performance of multiple PMUTs can be obtained in parallel by multiplying the multiplier on this basis. According to Equations (7) and (8), the underwater acoustic receiving sensitivity of a single PMUT is 0.379 mV/Pa when the frequency is 600 kHz. For four PMUTs that are electrically connected in parallel, the underwater acoustic receiving sensitivity is 1.516 mV/Pa (−176.38 dB), which is close to the measured result of −173.9 dB.

**Figure 13:**
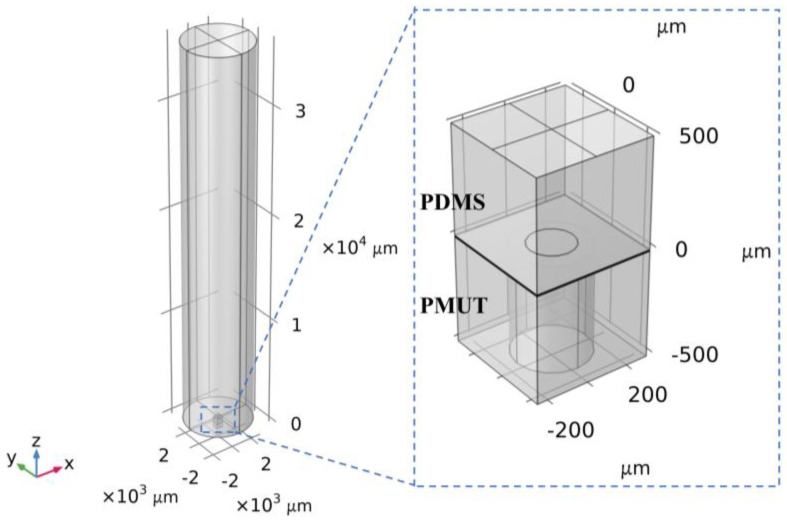
Simulation model of PMUT acoustic characteristics considering residual stress.

**Figure 14:**
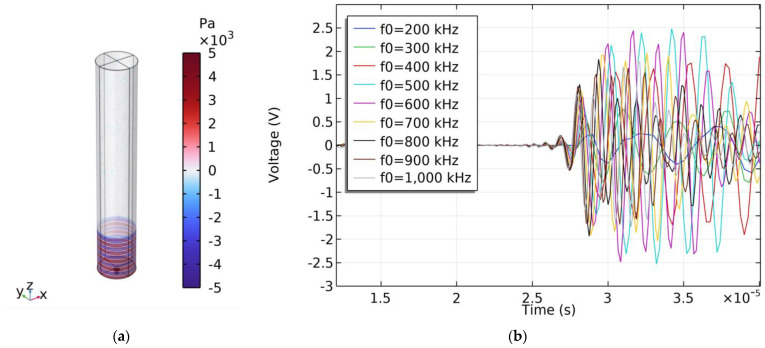
PMUT acoustic receiving simulation results. (**a**) Sound pressure distribution. (**b**) Echo signals with frequencies ranging from 200 kHz to 1000 kHz.

The model for the simulation of the acoustic characteristics of a single PMUT is shown in Figure 13, where the thickness of the PDMS is 0.5 mm, and the diameter and the height of the cylindrical water domain are 6 mm and 36 mm, respectively. According to the measured results in Figure 8 and Figure 9, the residual tensile stress of the AlN layer is set to be 500 MPa. To predict the acoustic characteristics of the PMUT, frequency sweeping is performed from 200 kHz to 1 MHz in 100 kHz steps. As shown in Figure 14a, the sound pressure of the sound wave propagating to the surface of the PMUT is about 10.6 kPa. The received signals of the PMUT after a 60 dB amplification at different frequencies are shown in Figure 14b. When the frequency is 600 kHz, the peak-to-peak voltage of the received signal after a 60 dB amplification is about 5 V. The receiving sensitivity of a single PMUT is calculated to be −186.53 dB. Since the number of PMUTs is 4, the receiving sensitivity of the PMUTs at 600 kHz is −174.48 dB.

**Figure 15:**
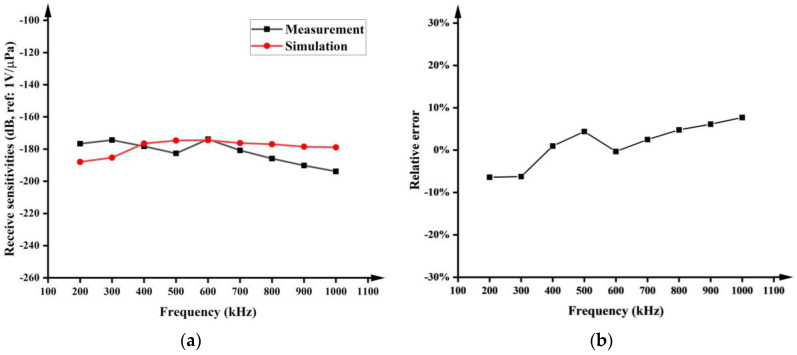
Simulation and measurement results of PMUTs’ underwater receiving sensitivity (**a**) and relative error (**b**).

Figure 15a shows the curve of the PMUT’s underwater receiving sensitivity against frequency. The optimum underwater receiving sensitivity obtained in the simulations is −174.48 dB (600 kHz, amplified by 60 dB), which only shows a small deviation of 0.33% compared to the measured value of −173.9 dB (600 kHz, amplified by 60 dB). Figure 15b shows the relative error between the simulated and measured results of the receiving sensitivity of the PMUTs at different frequencies, where the maximum relative error is ±7%. The good agreement between the simulation and measurement indicates that the PMUT simulation model can accurately predict the receiving sensitivity of the PMUT. The theoretical underwater resonant frequency of 310.5 kHz was obtained with Equations (1)–(4), which was also identified in the test, as shown in Figure 15a, but it was not identified by the simulation, meaning improvements are still needed for the simulation. In addition, the peak of the receiving sensitivity at 600 kHz identified by both the simulation and test results may be caused by the coupling effect among the liquid, PDMS, and PMUTs [23].

Table 2 compares the PMUTs reported in recent years with resonant frequencies in the range of 400 kHz to 1 MHz. The −6 dB bandwidth of the PMUTs presented herein is about 500 kHz, which achieves a bandwidth enhancement of at least two-fold. The receiver sensitivity per unit area after removing the amplifier’s gain is 1.37 V/μPa/mm^2^. The performances of the proposed PMUTs in this paper are superior to those of the currently reported PMUTs, with a nearly double improvement. The receiving sensitivity, bandwidth, and receiving angles of the PMUTs are on par with those of BENTHOWAVE INSTRUMENT INC.’s bulk ultrasonic transducers, BII-7630 and BII-7690. This confirms the viability of AlN PMUTs for preliminary use in 3D sonar imaging.

## 6. Conclusions

In this paper, a 2 × 2 AlN PMUT with an optimized ratio of 0.6 for the piezoelectric layer diameter to the backside cavity diameter is designed and fabricated for sonar imaging. The characterization test result of the sample demonstrates a −6 dB bandwidth of approximately 500 kHz and a measured receiving sensitivity per unit area of 1.37 V/μPa/mm^2^, which shows that it significantly surpasses the performance of previously reported PMUTs. Furthermore, a simulation model considering residual stress in the acoustic characteristics was established. The optimum underwater receiving sensitivity obtained in the simulations is −174.48 dB (600 kHz), which shows only a small deviation of 0.33% compared to the measured value of −173.9 dB (600 kHz). Moreover, the relative error between the simulated and measured underwater receiver sensitivities remains within ±7% across the frequency range of 200 kHz to 1000 kHz compared to the experimental data. This proves that the simulation is able to predict the underwater acoustic performance, providing a theoretical method for enhancing the uniformity of AlN PMUTs in subsequent steps during packaging and assembly.

## Figures and Tables

**Table 1:** Material parameters and dimensions of each layer.

Materials	Density (kg/m^3^)	Poisson’s Ratio	Young’s Modulus (GPa)	Thickness (μm)
Si	2330	0.28	170	3
Mo	10,200	0.31	314	0.2
AlN	3600	0.24	340	2
SiO_2_	2200	0.17	60	1

**Table 2:** A comparison between the fabricated PMUTs presented in this paper and previously reported PMUTs in the literature.

Materials	UW [24]	ZJU [8]	WHU [1]	This Work
Cavity size (μm)	460	280	500	345
Geometry	square	circular	hexagonal	circular
Array	3 × 3	10 × 10	7 × 8	2 × 2
Resonant frequency (kHz, in air)	600	986	438.62	767.2
Piezoelectric material	AlN	AlN	Sc_0.2_Al_0.8_N	AlN
Receiving sensitivity (dB) (Amplifier gain)	−237.39 (0 dB)	−178 (40 dB)	−173.4 (40 dB)	−173.9 (60 dB)
Receiving sensitivity (dB) ^1^	−237.39	−218	−213.4	−233.9
Receiving sensitivity per unit area (V/µPa/mm^2^) ^1^	0.71	0.53	0.58	1.37
−6 dB bandwidth (kHz)	-	~1.5	~68	~500

^1^ Without amplifier gain.

## Data Availability

All data, models, and code generated or used during the study appear in the submitted article.
